# Correction: Rhodium-catalyzed cycloaddition of carbonyl ylides for the synthesis of spiro[furo[2,3-*a*]xanthene-2,3′-indolin]-2′-one scaffolds

**DOI:** 10.1039/c7ra90114c

**Published:** 2018-01-15

**Authors:** B. V. Subba Reddy, E. Pravardhan Reddy, B. Sridhar, Y. Jayaprakash Rao

**Affiliations:** Centre for Semiochemicals, CSIR-Indian Institute of Chemical Technology Hyderabad 500 007 India basireddy@iict.res.in; Department of Chemistry, Osmania University Hyderabad-500 007 India; Laboratory of X-ray Crystallography, CSIR-Indian Institute of Chemical Technology Hyderabad 500 007 India; Department of Chemistry, Telangana University Nizamabad-500 007 India

## Abstract

Correction for ‘Rhodium-catalyzed cycloaddition of carbonyl ylides for the synthesis of spiro[furo[2,3-*a*]xanthene-2,3′-indolin]-2′-one scaffolds’ by B. V. Subba Reddy *et al.*, *RSC Adv.*, 2016, **6**, 50497–50499.

In the original manuscript, an incorrect CCDC number was given for the crystallographic data, and incorrect crystal structure data was provided as Electronic Supplementary Data for the manuscript. The correct CCDC number is 1451304 and the corresponding crystal structure data CIF file has been corrected for the original manuscript.

The Royal Society of Chemistry apologises for these errors and any consequent inconvenience to authors and readers.

## Supplementary Material

